# Impact of the COVID-19 pandemic on obsessive-compulsive symptoms in the Swiss general population

**DOI:** 10.3389/fpsyg.2023.1071205

**Published:** 2023-06-20

**Authors:** Johanna Otte, Nathalie Schicktanz, Dorothée Bentz

**Affiliations:** ^1^Division of Cognitive Neuroscience, Faculty of Psychology, University of Basel, Basel, Switzerland; ^2^Transfaculty Research Platform Molecular and Cognitive Neurosciences (MCN), Faculty of Psychology, University of Basel, Basel, Switzerland; ^3^Centre for Chronobiology, Psychiatric Hospital of the University of Basel, Basel, Switzerland

**Keywords:** obsessive-compulsive disorder (OCD), Coronavirus (COVID-19), OCD subtypes, fear of contamination, Obsessive-Compulsive-Inventory-Revised (OCI-R), stress

## Abstract

**Background:**

In the early stages of the COVID-19 pandemic, mental-health experts called attention to a possible deterioration of obsessive-compulsive symptoms (OCSs). In particular, people suffering from a fear of contamination were considered a vulnerable population.

**Aim:**

The aim of this study was to investigate the change in OCSs from before to during the pandemic within the Swiss general population, and to examine a possible relationship of OCSs to stress and anxiety.

**Methods:**

This cross-sectional study was implemented as an anonymized online survey (*N* = 3,486). The Obsessive-Compulsive Inventory-Revised (OCI-R) was used to assess global OCS severity (range: 0–72, clinical cut-off > 18) and specific OCS dimensions (range: 0–12) during the second wave of the pandemic and retrospectively for before the pandemic. Participants were asked to report stress and anxiety in the previous 2 weeks before the survey.

**Results:**

Participants reported significantly higher OCI-R total scores during (12.73) compared to before the pandemic (9.04, mean delta increase: 3.69). Significantly more individuals reported an OCI-R total score exceeding the clinical cut-off during (24%) than before the pandemic (13%). OCS severity increased on all symptom dimensions, but was most pronounced on the washing dimension (all with *p* < 0.001). Self-reported stress and anxiety were weakly associated with differences in severity in total score and symptom dimensions (with *R*^2^ < 0.1 and *p* < 0.001).

**Conclusion:**

Our results indicate that the full spectrum of people with OCS should be considered as risk groups for symptom deterioration during a pandemic and when assessing its possible long-term effects of such.

## 1. Introduction

With the emergence and spread of COVID-19, countries all over the world faced new challenges. To prevent its spreading, governments implemented measures such as lockdowns, quarantines, medical face masks, and contact restrictions. The measures that were intended to prevent infections affected almost all areas of life. It is therefore important to assess not only the physiological consequences of COVID-19 infections but also the psychological impact of the COVID-19 pandemic and its preventive measures responding to it.

One group of people who might have been particularly at risk of experiencing deteriorating mental health conditions were those with obsessive-compulsive symptoms (OCSs). Retrospective data has indicated a connection between changes in activities or routines and disease progression (Coles et al., 2011). In the early stages of the pandemic, mental-health experts drew attention to a possible deterioration of OCSs (Banerjee, 2020; Rivera and Carballea, 2020).

Around 0.7–2.3% of the general population fulfill the DSM criteria for clinically relevant obsessive-compulsive disorder (OCD); around 5% qualify for subthreshold OCD and fulfill some but not the complete set of the DSM criteria; and as much as 8% of the general population have OCSs without fulfilling any core symptoms of the DSM criteria (Angst et al., 2004; Ruscio et al., 2010; Adam et al., 2012). Affected individuals either attempt to ignore or suppress urges and thoughts (obsessions) by means of avoidance behavior or to neutralize them by performing repetitive behavior, such as handwashing (compulsions) to prevent or reduce distress (American Psychiatric Association [APA], 2013). One of the most prevalent subtypes of OCD is contamination OCD (C-OCD), which comprises around 56% of patients with OCD (Mataix-Cols et al., 2003). Obsessions of people with C-OCD are about fear of contamination (themselves, others). C-OCD is often accompanied by the avoidance of certain triggers (e.g., not touching certain objects or surfaces) and/or compulsive cleaning and washing (Veale and Roberts, 2014).

It is conceivable that governmental measures to limit the spread of COVID-19 could have especially triggered obsessions and compulsions characteristic of C-OCD, such as the fear of contamination and excessive cleaning, leading to an increase of symptoms related to C-OCD. Anecdotal evidence (French and Lyne, 2020), cross-sectional self-report data on C-OCD (Abba-Aji et al., 2020; Hassoulas et al., 2021) and OCD (Wheaton et al., 2021), and longitudinal data on patients with C-OCD (Jelinek et al., 2021c) and OCD (Liao et al., 2021) have indicated that there was a heightening of C-OCD related symptomatology during the pandemic. In addition, a study on a clinical population receiving psychiatric care prior to COVID-19 suggested that the presence of C-OCD before the pandemic was a risk factor for the exacerbation of OCD symptomatology during the pandemic (Davide et al., 2020).

However, there is also evidence that the impact was not only limited to C-OCD-related symptomatology and that severity increased on all measured symptom dimensions, as shown by longitudinal data on patients with OCD (Benatti et al., 2020, 2022) that examined an Italian outpatient population during the first (Benatti et al., 2020) and second (Benatti et al., 2022) pandemic waves, and by cross-sectional self-report data on OCD (Jelinek et al., 2021b; Wheaton et al., 2021).

While the exacerbation of C-OCD is intuitively comprehensible, an exacerbation of all obsessions and compulsions is also conceivable, considering that environmental factors such as stress and stressful or traumatic events are believed to play a role in the development and maintenance of OCD. Stress can be conceptualized as the occurrence of a challenge that requires adaptation (McEwen, 1998). Stressful life events (SLEs) refer to events that require readjustment, such as illness or unemployment. In general, SLEs are accompanied by feelings of stress and anxiety, which in turn may contribute to the onset and exacerbation of preexisting disorders. Uncertainty about how and when people will be able to return to normal routines over a prolonged period can be considered as a chronic stressor to vulnerable individuals. Chronic stress leads to behavioral inflexibility and overreliance on habitual strategies, which are known behavioral impairments in patients with OCD (Morgado et al., 2013; Raposo-Lima and Morgado, 2020). Furthermore, a cross-sectional study showed that self-reported stress by OCD patients is positively correlated with OCD severity (Morgado et al., 2013).

In several studies, patients with OCD have reported that the onset of the disorder coincided with or was preceded by stressful or traumatic life events (Lensi et al., 1996; Real et al., 2011; Rosso et al., 2012; Kracker Imthon et al., 2020), by changes in routines, or by general stress (Coles et al., 2011). In line with this assumption, a study found that 60% of participants with OCD had at least one SLE in the year before the onset of the disorder (Rosso et al., 2012). The Swiss Corona Stress Study (de Quervain et al., 2020b) indicated that feelings of stress, depression, and anxiety increased in Switzerland during the pandemic. The main drivers of psychological stress included burdens due to pandemic-related changes in work, school, or education, financial losses, or a fear that someone close is ill. Problems that resemble those named and classified as SLE in cross-sectional studies have preceded the development of OCD in some people affected by OCD (Real et al., 2011; Kracker Imthon et al., 2020). In any case, it is possible that not only individuals who had OCSs before they experienced a deterioration of such symptoms but also that individuals who were asymptomatic before the pandemic may have developed obsessive-compulsive maladaptive behaviors during it. It is therefore especially important to investigate not only clinical and subclinical populations with OCSs but also to obtain more information on the development of symptoms during the pandemic in the general population, as diagnosis and treatment as early as possible are critical to preventing OCD from becoming chronic (Fineberg et al., 2019).

The aim of this study was to investigate the change in OCS severity from before to during the COVID-19 pandemic within a large sample from the Swiss general population and to examine the relationship of stress and anxiety with OCSs. First, we were interested in the change of severity across all symptom dimensions and in whether the number of people within the sample reporting clinically relevant symptoms increased compared to before the pandemic. Second, we were interested in the change of severity for six different symptom dimensions (washing, checking, ordering, obsessing, hoarding, and neutralizing) and in whether there was a dimension that changed more than the others. Furthermore, we investigated the association of OCSs with stress and anxiety. The following six hypotheses were tested: (1) The severity of OCSs increased compared to before the pandemic. (2) Symptom severity increased across all symptom dimensions. (3) The increase in symptom severity did not differ between the symptom dimensions. (4) The number of people reporting clinically relevant OCSs increased during the pandemic compared to before it. (5) The increase in symptom severity was associated with feelings of stress. (6) The increase in symptom severity was associated with feelings of anxiety.

## 2. Methods

### 2.1. Design, setting, participants

This cross-sectional study was implemented as an anonymized online survey. It was attached to the follow-up of the Swiss Corona Stress Study (de Quervain et al., 2020a), which investigated subjective well-being during the COVID-19 pandemic, especially the associations between psychological stress and the COVID-19 pandemic in the Swiss population. Participants were recruited via a media release of the University of Basel, local newspapers, radio interviews, and social media. The inclusion criteria for the Swiss Corona Stress Study were Switzerland as country of residence and an age over 14. Participation in the previous Swiss Corona Stress Study (de Quervain et al., 2020b) during the first pandemic wave was an exclusion criterion. Of the 11,612 participants from all regions of Switzerland taking part in the Swiss Corona Stress Study (de Quervain et al., 2020a), 3,655 voluntarily continued with the study on OCSs. As we were interested in OCSs in an adult population, we only included participants between 18 and 65 years old in our analysis (*N* = 3,486). Data was collected from November 11 to 19, 2020, during the second pandemic wave in Switzerland, when infection numbers were high. At that time, there were measures such as restrictions on public and private gatherings, a recommendation for home office, obligations to wear masks, and quarantine in cases of infection. It was not necessary to acquire approval of the study protocol from the local ethics committee, as the survey was anonymized. Informed consent from participants was provided prior to study participation.

### 2.2. Procedure and outcomes

The anonymized online survey was divided into two parts. The first part consisted of the Swiss Corona Stress Study (de Quervain et al., 2020a) and the second part of the additional study on OCSs. Interested people visited a website^1^ that was available in three of Switzerland’s national languages, namely in German, French, and Italian. The software SoSci Survey (SoSci, 2022) was used to implement the survey and recorded the day of participation but no IP addresses or timestamps. The first part took about 15 min to complete and the second part 10 min. All items required a response for the survey to be completed.

After providing general study information and acquiring informed consent, the Swiss Corona Stress Study collected sociodemographic data including gender, age, nationality, education, profession, and preexisting psychiatric conditions (including OCD). Among other things, participants were then asked to rate their stress and anxiety levels in the two previous weeks on two 6-point Likert scales, ranging from 0 (not at all) to 5 (extremely strong), with higher scores indicating higher levels of stress or anxiety.

Participants could then choose to end participation in the survey or to continue with the second part, the survey on OCSs during the pandemic, which formed the basis of this report. OCSs were assessed with the Obsessive-Compulsive Inventory-Revised (OCI-R) questionnaire, using the German (Gönner et al., 2007), French (Zermatten et al., 2006), and Italian (Marchetti et al., 2010) versions.

Participants first filled out the OCI-R for the previous month to assess current OCSs (during the pandemic). The OCI-R instructions consisted of German, French, and Italian translations of the following two sentences: “The following statements describe behaviors or experiences that many people exhibit in their everyday lives. Please mark the number that best describes how much you were affected by or suffered from a behavior or experience in the past month.” Afterward they filled out a second OCI-R. This one was for the corresponding month exactly one year ago to retrospectively assess subjective OCSs from before the pandemic (prepandemic). For this second OCI-R, instructions were given as German, French, and Italian translations of the following sentence: “Please mark the number that best describes how much you were affected by or suffered from a behavior or experience a year ago (during a month).”

The OCI-R is an 18-item self-rating questionnaire to evaluate obsessions and compulsions on six symptom dimensions, namely washing, checking, ordering, obsessing, hoarding, and neutralizing. Each dimension is measured by three items, which are rated on 5-point scales ranging from 0 (not at all) to 4 (extremely), higher scores indicating higher symptom severity. The OCI-R foresees the calculation of a total score (OCI-R total score) from the scores of all 18 items (ranging from 0 to 72, a higher total score indicating higher overall severity) and the calculation of six scores for each dimension (OCI-R washing, checking, ordering, obsessing, hoarding, neutralizing) from the three items belonging to the dimension in question (ranging from 0 to 12, higher scores indicating more severe symptoms on the specific dimension). The OCI-R was designed for use in clinical and non-clinical samples and differentiates between healthy people and patients with OCD at a cutoff of 18 with a Youden’s index of *J* = 0.66 (Gönner et al., 2009). At this threshold, 84% of patients with OCD and 82% of healthy people were categorized correctly (Gönner et al., 2009).

At the end of the study, participants received automated recommendations for stress reduction based on the information they provided in the first part of the survey. If they had also completed the second part, they additionally received general information on OCD, treatment options, and contact addresses for help.

### 2.3. Statistical analyses

The data were analyzed in RStudio, version 1.4.1717 (Rstudio, 2022).

To analyze if a total OCI-R score increased significantly during the pandemic compared to before it, a paired *t*-test (one-sided) was calculated. The variable of interest was the total OCI-R score. To further investigate if the increase in symptom severity differed meaningfully between the six OCI-R dimensions (OCI-R washing, checking, ordering, obsessing, hoarding, neutralizing), a linear mixed-effects analysis was conducted, using the lme4 package for R (Bates et al., 2015). Time (prepandemic, during pandemic) and the six OCI-R dimensions were included as fixed effects with an interaction term. Intercepts for subjects were added as random factors. Significance was assessed by a likelihood-ratio test of the full model (including the interaction between time and dimension) against a null model (without the interaction). In case of a significant interaction, a *post-hoc* test was performed for each subdimension separately. For each, a one-sided paired *t*-test was calculated to analyze differences in the scores from prepandemic and during the pandemic on the corresponding dimension. Due to the seven variables of interest, the significance threshold was set to *p* < 0.007. Cohen’s *dz* is reported as the effect-size measurement. According to the conventions (Cohen, 1988), *dz* ≥ 0.8, *dz* ≥ 0.5, and *dz* ≥ 0.2 were considered large, intermediate, and small effects, respectively.

To analyze if the percentage of participants with clinically relevant OCI-R symptoms changed from prepandemic to during the pandemic, we first classified each OCI-R total score as either under or over the clinical threshold of 18 (Gönner et al., 2009) for both prepandemic and during the pandemic. Scores under 18 were categorized as not clinically relevant OCSs and scores above 18 were categorized as clinically relevant OCSs. Second, with McNemar’s chi-square tests (χ^2^) we analyzed whether the number of people reporting clinically relevant OCSs had increased. Then a phi coefficient (Φ) was calculated as the effect size. The conventions for interpreting Φ were: Φ = 0.1 was considered a small effect; Φ = 0.3 a medium effect, and Φ = 0.5 a large effect (Cohen, 1988). We provide the percentage of participants in the sample reporting clinically relevant symptoms prepandemic and the percentage reporting clinically relevant symptoms during the pandemic, χ^2^ values, the associated *p*-values, and effect size Φ.

Linear regression was used to assess the association of current stress and anxiety levels with OCSs. Stress ratings and anxiety ratings were used as independent variables in separate models. The differences (scores during the pandemic minus prepandemic), for the OCI-R totals as well as for the six symptom dimension scores (OCI-R washing, checking, ordering, obsessing, hoarding, and neutralizing), were used as dependent variables in separate linear models. We report B, standard error of B, t with *p*-values, *R*^2^, and *F* with *p*-values for each linear model in two separate tables for stress and anxiety levels.

## 3. Results

### 3.1. Demographics

Of the 11,612 participants in the Swiss Corona Stress Study, 3,486 (30%) met the inclusion criteria for the additional study on OCSs and completed the corresponding survey. In this subsample, 74.2% of participants reported female gender, 25% male, 0.3% diverse, and 0.4% gave no response. The mean age of participants was 37.9 years (*SD* = 12.1; range = 18–65). Regarding their place of residence, 61.6% lived in the German-speaking cantons of Switzerland, 27% in the French-speaking cantons, and 11.4% in the Italian-speaking canton. The German version of the questionnaire was chosen by 56.3% of participants, the French version by 24.1%, and the Italian version by 19.7%. Regarding their highest education levels, 17.8% had completed vocational school and an apprenticeship, 49% a university degree, 5% a doctorate, 22.7% upper secondary school, and 2.7% mandatory education. Regarding their current positions, 68.7% were currently employed, 13.3% were studying at a university, 4.4% were going to school or doing an apprenticeship, 4.6% were looking for a job, 1.3% were receiving a pension, and 2.2% were receiving a disability pension. Of all the participants, 32.4% reported having at least one preexisting psychiatric disorder before the pandemic started, and depression (18.5%) and anxiety disorders (17.8%) were the most prevalent. Regarding OCD, 3.8% of participants reported to have been diagnosed with OCD prior to the pandemic. These participants indicated a mean prepandemic OCI-R total of 21.05 (*SD* = 14.20), compared to 8.56 (*SD* = 8.54) for the participants who indicated that they had not been diagnosed with OCD before the pandemic.

### 3.2. Increase of OCI-R scores during the pandemic compared to prepandemic

The one-sided paired *t*-test indicated that OCI-R total scores increased significantly during the pandemic compared to prepandemic (during: *M* = 12.73; *SD* = 10.63, pre: *M* = 9.04, *SD* = 9.15; *t* (3318) = −36, *p* < 0.001, *dz* = 0.62).

To investigate if all six OCI-R dimensions increased comparably during the pandemic, a linear mixed effects model was calculated with an interaction between time (prepandemic, during pandemic) and OCI-R dimensions (OCI-R washing, checking, ordering, obsessing, hoarding, neutralizing). A likelihood-ratio test comparing the full model (including the interaction) to a null model (without the interaction) showed that the interaction between the two factors time and dimension was significant [χ^2^(5) = 340, *p* < 0.001].

To describe the interaction, paired *t*-tests were run as *post-hoc* tests for each dimension separately. These revealed significant increases in symptoms for all the dimensions (see Table 1). However, the dimension washing increased the most with a medium effect size of *dz* = 0.57, followed by obsessing (dz = 0.45).

**TABLE 1:** Mean OCI-R symptom dimension scores prepandemic and during the pandemic with effect sizes.

Dimension OCI-R …	Prepandemic mean ± *SD*	During pandemic mean ± *SD*	*t*	*dz*	CI lower; upper
Washing	1.00 ± 2.00	2.27 ± 2.85	−33*	0.57	0.532; 0.603
Obsessing	2.26 ± 2.92	3.21 ± 3.31	−26*	0.45	0.42; 0.49
Checking	1.19 ± 2.08	1.75 ± 2.35	−23*	0.40	0.367; 0.436
Ordering	2.11 ± 2.79	2.70 ± 2.96	−21*	0.36	0.326; 0.395
Neutralizing	0.63 ± 1.50	0.91 ± 1.75	−16*	0.28	0.243; 0.311
Hoarding	1.85 ± 2.51	2.03 ± 2.51	−6.9*	0.12	0.086; 0.153

OCI-R, Obsessive-Compulsive Inventory-Revised; CI, Confidence interval; *dz* = Cohen’s *d*.

*Significant after Bonferroni correction (significance threshold *p* < 0.008).

### 3.3. Change in percentage of clinically relevant symptoms prepandemic and during the pandemic

Based on the subjective recollection of the participants, 13% of participants were classified as having clinically relevant OCSs prepandemic, compared to 24% during the pandemic (i.e., OCI-R total score > 18). The McNemar’s test indicated that the percentage of participants reporting clinically relevant OCSs during the pandemic significantly increased compared to prepandemic [χ^2^ (1) = 300, *p* < 0.001]. The corresponding effect size Φ = 0.62 indicates a large effect.

### 3.4. Associations of current stress and OCS severity

All seven univariate linear-regression models that analyzed the effect of current stress levels on changes in symptom severity for the OCI-R total score and for the six symptom dimensions (OCI-R washing, checking, ordering, obsessing, hoarding, and neutralizing) were significant with *p* < 0.001, but with a small *R*^2^ < 0.1. For exact *R*^2^ values, see Table 2.

**TABLE 2:** Associations of differences in OCI-R symptom dimensions with current stress.

Dimension OCI-R …	*B*	*SE*(*B*)	*t*	*R* ^2^	*F*(*df*)
Washing	0.22	0.02	9.52*	0.027	90.7(1;3317)*
Checking	0.17	0.01	12.20*	0.043	149(1;3317)*
Ordering	0.16	0.02	10.14*	0.030	103(1;3317)*
Obsessing	0.39	0.02	18.85*	0.097	355(1;3317)*
Hoarding	0.09	0.01	6.29*	0.012	39.6(1;3317)*
Neutralizing	0.07	0.01	7.07*	0.015	50(1;3317)*
OCI-R total	1.10	0.06	18.70*	0.096	350(1;3317)*

OCI-R, Obsessive-Compulsive Inventory-Revised; B, regression coefficient; SE, standard error.

*Significant (significance threshold *p* < 0.001).

### 3.5. Associations of current anxiety and OCS severity

All seven univariate linear regression models that analyzed the effect of current anxiety levels on changes in symptom severity for the OCI-R total score and for the six symptom dimensions (OCI-R washing, checking, ordering, obsessing, hoarding, and neutralizing) were significant with *p* < 0.001, but with a small *R*^2^ < 0.1 (total score *R*^2^ = 0.115). For exact *R*^2^ values, see Table 3.

**TABLE 3:** Associations of differences in OCI-R symptom dimensions with current anxiety.

Dimension OCI-R …	*B*	*SE*(*B*)	*t*	*R* ^2^	*F*(*df*)
Washing	0.33	0.02	15.67*	0.069	245(1;3317)*
Checking	0.16	0.01	12.73*	0.047	162(1;3317)*
Ordering	0.15	0.02	9.92*	0.029	98.5(1;3317)*
Obsessing	0.35	0.02	17.9*	0.088	321(1;3317)*
Hoarding	0.08	0.01	5.48*	0.009	30.1(1;3317)*
Neutralizing	0.07	0.01	7.25*	0.016	52.6(1;3317)*
OCI-R total	1.13	0.05	20.8*	0.115	433(1;3317)*

OCI-R, Obsessive-Compulsive Inventory-Revised; B, regression coefficient; SE, standard error.

*Significant (significance threshold *p* < 0.001).

## 4. Discussion

In a large sample of 3,486 participants from the Swiss general population, we found a significant increase in self-reported OCSs during the second wave of the COVID-19 pandemic compared to retrospectively assessed OCSs. All six OCI-R dimension scores increased significantly during the pandemic. In comparison to their recollections of before the pandemic, a significantly higher proportion of participants reported an OCI-R total score exceeding the clinical threshold of 18. The increase in total OCI-R score and the six dimensions were significantly, but weakly, associated with self-reported stress and anxiety levels regarding the 2 weeks prior to the study.

The finding that during the COVID-19 pandemic multiple types of obsessions and compulsions, and not only fear of contamination, were affected in the general population corresponds to previous findings that have also found OCS severity to have increased generally and across different OCS dimensions (Jelinek et al., 2021b; Khosravani et al., 2021; Wheaton et al., 2021). However, the magnitude of change varied by symptom dimension in our study, washing showing the nominally largest effect size. This was in line with studies that showing that individuals with C-OCD were especially affected by the COVID-19 pandemic (Abba-Aji et al., 2020; Hassoulas et al., 2021; Jelinek et al., 2021c; Wheaton et al., 2021).

The association between the increase in total OCI-R score and the six dimensions with self-reported stress and anxiety levels regarding the 2 weeks prior to the study is in line with another cross-sectional study (Abba-Aji et al., 2020), which showed an association between washing compulsions and stress, and with cross-sectional studies that have shown an association between stress and the occurrence and severity of overall OCD symptoms (Morgado et al., 2013; Tandt et al., 2021). However, our correlational design does not allow any causal inferences, as stress and anxiety might also be the consequence of a worsening of OCD symptomatology. To further evaluate the association of stress and anxiety with OCD, carefully conducted prospective longitudinal studies are needed. In further studies, stress and anxiety should at best be experimentally manipulated or at least investigated using validated questionnaires to measure chronic stress and anxiety [e.g., the TICS (Schulz et al., 2004) and STAI (Spielberger et al., 1970)].

If one assumed that stress and anxiety do causally exacerbate OCSs, there would still be the question of why we found only a weak association in our population between OCD and stress and anxiety. One interpretation is that additional factors other than stress and anxiety might be of higher relevance and have to be further scrutinized. The chronicity of symptoms might be an important factor. For example, another cross-sectional study in Qatar showed that those with a shorter illness duration (<10 years) experienced a larger deterioration of OCSs (Siddiqui et al., 2022). One can speculate that people with a more chronic symptomatology are less prone to external changes, are better embedded in a treatment, or are on stable psychopharmacological medication. The later assumption is in line with a study that showed that patients with OCD (including patients with contamination and illness obsession) who were already in treatment (exposure and response prevention/ERP, psychopharmacological) before the pandemic did not suffer from a worsening of symptoms during the pandemic (Carmi et al., 2021). Another important factor seems to be a prepandemic OCD diagnosis (Jelinek et al., 2021a). The first factor was not queried in our study, and the existence of a prepandemic OCD diagnosis relied only on self-reporting and was not verified by a clinician. Another interpretation of the weak association would be that the obsessive-compulsive behavior is actually practiced as a maladaptive coping mechanism to deal with pandemic stress and anxiety. If that were the case, it would explain why we only find a small association, since the OCD behavior would be used to control excessive stress and anxiety.

Interestingly, the largest association was found between stress and anxiety and the OCI-R dimension obsessions. A closer look at the three items measuring this dimension reveals that they measure quite unspecifically unpleasant thoughts (intrusions) and not specific obsessions. This may lead to overreporting when the OCI-R is administered in times of real threat such as the pandemic, which could particularly affect those who are stressed and anxious. Individuals who felt exceptionally stressed and anxious during the COVID-19 pandemic might have had more unpleasant pandemic-associated thoughts that do not necessarily qualify as obsessions despite being detected as such in the OCI-R. This might account for the highest correlation between stress and anxiety and that OCI-R dimension. Further studies should investigate how to improve methods to better differentiate between obsessions and event-specific thoughts that can be considered normal reactions to real threats.

Overall, the following limiting factors of our study design must be considered in interpreting our results: (1) Our study was not a representative survey and (2) prepandemic OCSs were collected retrospectively. Retrospective assessments are prone to recall bias with higher recall bias when more time lies between recall and the time that is recalled. There is a propensity to reconstruct memories to align them with current events and experiences (Wilhelm et al., 2004). Participants of our survey evaluated the past from their current perspective during the pandemic, which may have led to a recall bias. Both factors might result in an overestimation of the amount of people that experienced a worsening of existing OCSs and of the strength of deterioration of OCSs. (3) The study was not an experiment. This means that although the survey of stress and anxiety as well as of OCSs refers to successive time periods, no causal conclusions can be drawn about the relationship between OCSs and stress and anxiety. (4) Self-reported stress and anxiety were measured globally by a single item. More comprehensive information about the association between OCS with stress and anxiety might be achieved by using established questionnaires, such as the TICS (Schulz et al., 2004) to measure stress or the STAI (Spielberger et al., 1970) to measure anxiety. (5) The OCI-R is a valid and reliable tool for OCD and its symptom dimensions. However, it was created and validated before the pandemic. Thus, the 5-point Likert scale may not be sensitive enough for the washing dimension to differentiate adaptive behaviors exhibited during the pandemic that were recommended by health authorities as preventive measures from clinically relevant excessive behaviors. This may have potentially led to an overestimation of the true effect on the OCI-R washing score and on the OCI-R total score. (6) On the other hand, the use of the OCI-R total score to capture clinically relevant OCD may have also resulted in an underestimation of individuals whose symptoms transitioned to the clinical domain during the pandemic because the OCI-R is not designed to capture monosymptomatic patients who experience OCS on only one dimension. These individuals may have experienced a worsening of only one symptom dimension and so stayed below the cut-off in the total score. Taking these points into account, the retrospective OCI-R scores to measure OCS and OCD before the pandemic and during the pandemic should be interpreted with caution. Future studies should consider this and confirm the results found in this study with the gold standard for assessing OCSs and diagnosing OCD in clinical interviews within a longitudinal design, for example by using the YBOCS questionnaire (Goodman et al., 1989).

The strength of our study is clearly the number of people surveyed about OCSs. We are the first study to investigate a possible impact of the COVID-19 pandemic on OCSs in a large sample from the Swiss general population. We found that not only the washing dimension was negatively impacted, but also other OCSs such as ordering or checking, a consequence of the pandemic on the general population that appears less intuitive. Further, this study is the first to examine clinically relevant changes in OCSs in the Swiss general population during the pandemic. Guzick et al. (2021) discussed in their review, studies in other countries have reported similar results at various stages of the pandemic. But individuals that reached the clinical cut-off in our study at the time point of the measurement are not the only ones of relevance. OCSs are widely spread in the general population (Ruscio et al., 2010; Adam et al., 2012), and some individuals reporting OCSs will be diagnosed with OCD at some point in their lives (Fineberg et al., 2013). In order to prevent OCD from becoming chronic, early diagnosis and treatment are critical (Fineberg et al., 2019). In this context, our findings underscore the importance of taking into account the full spectrum of individuals with OCSs, instead of only people with a fear of contamination, as a risk group for deterioration in the aftermath of the COVID-19 pandemic. This should be considered when assessing risk groups for deterioration of symptoms during the COVID-19 pandemic or subsequent pandemics and in planning countermeasures.

## Data availability statement

The raw data supporting the conclusions of this article will be made available by the authors, without undue reservation.

## Ethics statement

Ethical review and approval was not required for the study on human participants in accordance with the local legislation and institutional requirements, because it was an anonymized online study. The participants provided their informed consent to participate in the study.

## Author contributions

DB and JO planned and directed the study and drafted the manuscript. NS implemented the online survey and gave statistical advice. JO performed the statistics. All authors contributed and gave final approval of the research report.

## Footnotes

1
www.coronastress.ch

## References

[B1] Abba-Aji A. Li D. Hrabok M. Shalaby R. Gusnowski A. Vuong W. (2020). COVID-19 pandemic and mental health: Prevalence and correlates of new-onset obsessive-compulsive symptoms in a Canadian province. *Int. J. Environ. Res. Public Health* 17:6986. 10.3390/ijerph17196986 32987764 PMC7579625

[B2] Adam Y. Meinlschmidt G. Gloster A. Lieb R. (2012). Obsessive-compulsive disorder in the community: 12-month prevalence, comorbidity and impairment. *Soc. Psychiatry Psychiatr. Epidemiol.* 47 339–349. 10.1007/s00127-010-0337-5 21287144

[B3] American Psychiatric Association [APA] (2013). *Diagnostic and Statistical Manual of Mental Disorders*, 5th Edn. Washington, DC: American Psychiatric Press.

[B4] Angst J. Gamma A. Endrass J. Goodwin R. Ajdacic V. Eich D. (2004). Obsessive-compulsive severity spectrum in the community: Prevalence, comorbidity, and course. *Eur. Arch. Psychiatry Clin. Neurosci.* 254 156–164. 10.1007/s00406-004-0459-4 15205969

[B5] Banerjee D. (2020). The other side of COVID-19: Impact on obsessive compulsive disorder (OCD) and hoarding. *Psychiatry Res.* 288:112966. 10.1016/j.psychres.2020.112966 32334276 PMC7151248

[B6] Bates D. Mächler M. Bolke B. Walker S. (2015). Fitting linear mixed-effects models using lme4. *J. Stat. Softw.* 67 1–48. 10.18637/jss.v067.i01

[B7] Benatti B. Albert U. Maina G. Celebre L. Girone N. Bramante S. (2022). First vs second wave of COVID-19 pandemic in patients with obsessive compulsive disorder: A multicentre report from tertiary clinics in Northern Italy. *J. Psychiatr. Res.* 154 293–299. 10.1016/j.jpsychires.2022.07.058 35964348 PMC9353706

[B8] Benatti B. Albert U. Maina G. Fiorillo A. Celebre L. Girone N. (2020). What happened to patients with obsessive compulsive disorder during the COVID-19 pandemic? A multicentre report from tertiary clinics in northern Italy. *Front. Psychiatry* 11:720. 10.3389/fpsyt.2020.00720 32793008 PMC7385249

[B9] Carmi L. Ben-Arush O. Fostick L. Cohen H. Zohar J. (2021). Obsessive compulsive disorder during Coronavirus disease 2019 (COVID-19): 2- and 6-month follow-ups in a clinical trial. *Int. J. Neuropsychopharmacol.* 24 703–709. 10.1093/ijnp/pyab024 34048557 PMC8195092

[B10] Cohen J. (1988). *Statistical Power Analysis for the Behavioral Sciences*, 2nd Edn. Hillsdale, NJ: L. Erlbaum Associates.

[B11] Coles M. Johnson E. Schubert J. (2011). Retrospective reports of the development of obsessive compulsive disorder: Extending knowledge of the protracted symptom phase. *Behav. Cogn. Psychoth.* 39 579–589. 10.1017/S135246581100004X 21392416

[B12] Davide P. Andrea P. Martina O. Andrea E. Davide D. Mario A. (2020). The impact of the COVID-19 pandemic on patients with OCD: Effects of contamination symptoms and remission state before the quarantine in a preliminary naturalistic study. *Psychiatry Res*. 291:113213. 10.1016/j.psychres.2020.113213 32535508 PMC7280119

[B13] de Quervain D. Aerni A. Amini E. Bentz D. Coynel D. Freytag V. (2020a). *The Swiss Corona Stress Study: Second pandemic wave, November 2020.* Available online at: https://osf.io/6cseh/ (accessed December 16, 2020).

[B14] de Quervain D. Aerni A. Amini E. Bentz D. Coynel D. Gerhards C. (2020b). *The Swiss Corona Stress Study.* Available online at: https://osf.io/jqw6a/ (accessed July 01, 2020).

[B15] Fineberg N. Dell’Osso B. Albert U. Maina G. Geller D. Carmi L. (2019). Early intervention for obsessive compulsive disorder: An expert consensus statement. *Eur. Neuropsychopharmacol.* 29 549–565. 10.1016/j.euroneuro.2019.02.002 30773387

[B16] Fineberg N. Hengartner M. Bergbaum C. Gale T. Gamma A. Ajdacic-Gross V. (2013). A prospective population-based cohort study of the prevalence, incidence and impact of obsessive-compulsive symptomatology. *Int. J. Psychiatry Clin. Pract.* 17 170–178. 10.3109/13651501.2012.755206 23205952

[B17] French I. Lyne J. (2020). Acute exacerbation of OCD symptoms precipitated by media reports of COVID-19. *Ir. J. Psychol. Med.* 37 291–294. 10.1017/ipm.2020.61 32434605 PMC7306547

[B18] Gönner S. Ecker W. Leonhart R. Foa E. Huppert J. Leiberg S. (2009). *Obsessive-Compulsive Inventory-Revised (OCI-R) Manual – Deutsche Adaption.* London: Pearson.

[B19] Gönner S. Leonhart R. Ecker W. (2007). The German version of the obsessive-compulsive inventory-revised: A brief self-report measure for the multidimensional assessment of obsessive-compulsive symptoms. *Psychother. Psychosom. Med. Psychol.* 57 395–404. 10.1055/s-2007-970894 17590836

[B20] Goodman W. Price L. Rasmussen S. Mazure C. Fleischmann R. Hill C. (1989). The yale-brown obsessive compulsive scale. I. Development, use, and reliability. *Arch. Gen. Psychiatry* 46 1006–1011. 10.1001/archpsyc.1989.01810110048007 2684084

[B21] Guzick A. Candelari A. Wiese A. Schneider S. Goodman W. Storch E. (2021). Obsessive-compulsive disorder during the COVID-19 pandemic: A systematic review. *Curr. Psychiatry Rep.* 23:71. 10.1007/s11920-021-01284-2 34613498 PMC8493778

[B22] Hassoulas A. Umla-Runge K. Zahid A. Adams O. Green M. Hassoulas A. (2021). Investigating the association between obsessive-compulsive disorder symptom subtypes and health anxiety as impacted by the COVID-19 pandemic: A cross-sectional study. *Psychol Rep.* 125 3006–3027. 10.1177/00332941211040437 34412543 PMC9578079

[B23] Jelinek L. Göritz A. Miegel F. Moritz S. Kriston L. (2021a). Predictors of trajectories of obsessive-compulsive symptoms during the COVID-19 pandemic in the general population in Germany. *Transl. Psychiatry* 11:323. 10.1038/s41398-021-01419-2 34045444 PMC8155650

[B24] Jelinek L. Moritz S. Miegel F. Voderholzer U. (2021b). Obsessive-compulsive disorder during COVID-19: Turning a problem into an opportunity? *J. Anxiety Disord*. 77:102329. 10.1016/j.janxdis.2020.102329 33190017 PMC7644184

[B25] Jelinek L. Voderholzer U. Moritz S. Carsten H. Riesel A. Miegel F. (2021c). When a nightmare comes true: Change in obsessive-compulsive disorder over the first months of the COVID-19 pandemic. *J. Anxiety Disord.* 84:102493. 10.1016/j.janxdis.2021.102493 34752943 PMC8590107

[B26] Khosravani V. Aardema F. Samimi Ardestani S. Sharifi Bastan F. (2021). The impact of the coronavirus pandemic on specific symptom dimensions and severity in OCD: A comparison before and during COVID-19 in the context of stress responses. *J. Obsessive Compuls. Relat. Disord.* 29:100626. 10.1016/j.jocrd.2021.100626 33520614 PMC7834974

[B27] Kracker Imthon A. Antônio Caldart C. do Rosário M. Fontenelle L. Constantino Miguel E. Arzeno Ferrão Y. (2020). Stressful life events and the clinical expression of obsessive–compulsive disorder (OCD): An exploratory study. *J. Clin. Med.* 9:3371. 10.3390/jcm9103371 33096706 PMC7590000

[B28] Lensi P. Cassano G. Correddu G. Ravagli S. Kunovac J. Akiskal H. (1996). Obsessive–compulsive disorder. *Br. J. Psychiatr.* 169 101–107. 10.1192/bjp.169.1.101 8818377

[B29] Liao J. Liu L. Fu X. Feng Y. Liu W. Yue W. (2021). The immediate and long-term impacts of the COVID-19 pandemic on patients with obsessive-compulsive disorder: A one-year follow-up study. *Psychiatry Res.* 306:114268. 10.1016/j.psychres.2021.114268 34837883 PMC8562016

[B30] Marchetti I. Chiri L. Ghisi M. Sica C. (2010). Obsessive-compulsive inventory-revised (OCI-R): Presentazione e indicazioni di utilizzo nel contesto Italiano. *Psicoter. Cogn. Comport.* 16 69–84.

[B31] Mataix-Cols D. Cullen S. Lange K. Zelaya F. Andrew C. Amaro E. (2003). Neural correlates of anxiety associated with obsessive-compulsive symptom dimensions in normal volunteers. *Biol. Psychiatry* 53 482–493. 10.1016/s0006-3223(02)01504-4 12644353

[B32] McEwen B. (1998). Stress, adaptation, and disease: Allostasis and Allostatic Load. *Ann. N. Y. Acad. Sci.* 840 33–44. 10.1111/j.1749-6632.1998.tb09546.x 9629234

[B33] Morgado P. Freitas D. Bessa J. Sousa N. Cerqueira J. (2013). Perceived stress in obsessive–compulsive disorder is related with obsessive but not compulsive symptoms. *Front. Psychiatry* 4:21. 10.3389/fpsyt.2013.00021 23565098 PMC3613755

[B34] Raposo-Lima C. Morgado P. (2020). The role of stress in obsessive-compulsive disorder: A narrative review. *Harvard Rev. Psychiatr.* 28 356–370. 10.1097/HRP.0000000000000274 33027102

[B35] Real E. Labad J. Alonso P. Segalàs C. Jiménez-Murcia S. Bueno B. (2011). Stressful life events at onset of obsessive-compulsive disorder are associated with a distinct clinical pattern. *Depress. Anxiety* 28 367–376. 10.1002/da.20792 21308889

[B36] Rivera R. Carballea D. (2020). Coronavirus: A trigger for OCD and illness anxiety disorder? *Psychol. Trauma* 12:S66. 10.1037/tra0000725 32496094

[B37] Rosso G. Albert U. Asinari G. Bogetto F. Maina G. (2012). Stressful life events and obsessive-compulsive disorder: Clinical features and symptom dimensions. *Psychiatry Res.* 197 259–264. 10.1016/j.psychres.2011.10.005 22370150

[B38] Rstudio (2022). *Share Everything R & Python Posit Connect.* Available online at: http://www.rstudio.com/ (accessed October 12, 2022).

[B39] Ruscio A. Stein D. Chiu W. Kessler R. (2010). The epidemiology of obsessive-compulsive disorder in the National Comorbidity Survey Replication. *Mol. Psychiatry* 15 53–63. 10.1038/mp.2008.94 18725912 PMC2797569

[B40] Schulz P. Schlotz W. Becker P. (2004). *TICS – Trierer Inventar Zum Chronischen Stress.* Göttingen: Hogreve-Verlag.

[B41] Siddiqui M. Wadoo O. Currie J. Alabdulla M. Al Siaghy A. AlSiddiqi A. (2022). The impact of COVID-19 pandemic on individuals with pre-existing obsessive-compulsive disorder in the state of Qatar: An exploratory cross-sectional study. *Front. Psychiatry* 13:833394. 10.3389/fpsyt.2022.833394 35492736 PMC9040606

[B42] SoSci (2022). *SoSci Survey GmBH. SoSci Survey - Professionelle Onlinebefragung Made in Germany.* Available online at: https://www.soscisurvey.de (accessed October 12, 2022).

[B43] Spielberger C. Gorsuch R. Lushene R. (1970). *Manual for the State-Trait Anxiety Inventory (Self-Evaluation Questionnaire).* Palo Alto, CA: Consulting Psychologists Press.

[B44] Tandt H. Debruyckere I. Leyman L. Colman R. De Jaeghere E. Van Parys H. (2021). How are OCD patients and family members dealing with the waxing and waning pattern of the COVID-19 pandemic? Results of a longitudinal observational study. *Psychiatr. Q.* 92 1549–1563. 10.1007/s11126-021-09932-9 34097247 PMC8182341

[B45] Veale D. Roberts A. (2014). Obsessive-compulsive disorder. *BMJ* 348:g2183. 10.1136/bmj.g2183 24709802

[B46] Wheaton M. Ward H. Silber A. McIngvale E. Björgvinsson T. (2021). How is the COVID-19 pandemic affecting individuals with obsessive-compulsive disorder (OCD) symptoms? *J. Anxiety Disord.* 81:102410. 10.1016/j.janxdis.2021.102410 33965747 PMC9759664

[B47] Wilhelm P. Schoebi D. Perrez M. (2004). Frequency estimates of emotions in everyday life from a diary method’s perspective: A comment on Scherer et al.’s survey-study “Emotions in everyday life.”. *Soc. Sci. Inf.* 43 647–665. 10.1177/0539018404047712

[B48] Zermatten A. Van der Linden M. Jermann F. Ceschi G. (2006). Validation of a French version of the obsessive–compulsive inventory-revised in a non-clinical sample. *Eur. Rev. Appl. Psychol.* 56 151–155. 10.1016/j.erap.2005.07.003

